# Diseases of the Ear, Nose and Throat

**Published:** 1910-06

**Authors:** P. Watson Williams


### DISEASES OF THE EAR, NOSE AND THROAT.

**Acute Labyrinthitis following Mastoid Operation.**—A variety of acute labyrinthitis not due to surgical traumatism is liable to arise after the radical mastoid operation, as in those grouped by Alexander under the term *labyrinthitis acuta circumscripta.* In the four instances reported by this observer the symptoms of labyrinthitis appeared within twenty-four hours after operation, viz. marked vertigo with vomiting and sometimes subjective movements of external objects, spontaneous nystagmus generally to the sound side, the patient lying on the sound side. After the rather violent onset, all the symptoms gradually subsided without any further operative interference, and at the end of a week the patients were perfectly well and able to get about. In two of the cases meningeal symptoms accompanied the labyrinthitis, but they disappeared even more rapidly than the labyrinthine symptoms. The author concludes that these manifestations were due to a transient serous labyrinthitis, and in all his four cases the recovery of the labyrinthine functions was complete.

Similar cases occur in the experiences of many operators, and now that vestibulotomy has become a practical procedure for suppurative labyrinthitis as well as for Ménière's disease, it is perhaps well to emphasise the transient character of the condition to which Alexander has directed attention.

Acute exacerbations of pre-existing and chronic labyrinthitis may of course follow a radical mastoid operation. These are cases in which the radical operation should be followed by vestibulotomy. Again, a traumatic breach of the external osseous wall in the course of a radical mastoid operation may be followed by acute labyrinthitis. These two classes of cases are due either to errors of omission or commission, unlike the form described by Alexander.[1]

[1] *Arch. f. Ohrenh.*, 1908, lxxv, 1; *J. Laryngol.*, 1910, xxv, 50.

**Cancer of the larynx of slow development.**—In its earlier stages cancer of the larynx is often very difficult to diagnose with certainty, and of any given neoplasm it is quite possible to remove a considerable fragment and yet on histological examination to find no indications of malignant disease. Some exceptional cases recently recorded must lead us to recast the prevailing view that malignant disease left to take its course is necessarily fatal within a relatively short period. Thus Harmon Smith[1] reports a case which in 1896 was shown by Gleitzmann to the American Laryngological Association as being probably malignant, he having first seen the patient towards the end of 1895. At that time a large snow-white mass covered the whole length of the right vocal cord. A piece submitted to the microscope showed that underlying epithelial cells showed marked proliferation, with a splitting of the nuclei; the submucosa showed a small-celled infiltration in consequence of connective tissue proliferation; the epithelial layer showed a tendency to invasion of the sub-epithelial tissues, as in carcinoma (Schwyzer). The case then disappeared from observation until August, 1907, *i.e.* almost twelve years from the time he was first seen. Then only certain areas of the white growth were to be seen, but the ventricular band was very much swollen, and parts of the cord on the opposite side were hidden by the similar swelling of the ventricular band on that side. There was no pain, no enlargement of glands, and no marked loss of weight. A piece then removed and submitted to Jno. Wright was reported to be probably pachydermia laryngis. In a second piece, removed a month later, the epithelium was found to dip down into the stroma in atypical fashion. There were several subsequent examinations of pieces removed from this growth from time to time. In January, 1908, the patient began to get worse and was weaker, and obstructive dyspnœa developed. The examination of the last piece removed in April, 1908, led Wright to the impression that the growth was now assuming a malignant histological appearance. Shortly after this the entire larynx was removed by Brewer, and Wright reported that a glance through the sections showed that the growth as a whole presented the characteristics of a flat-celled epithelioma. "We call it an epithelioma. Recognised fourteen years ago by two microscopists as being a malignant growth, it evidently has not changed its structure since." No external gland involvement was found.

Another interesting case with a very long-standing history was brought before the Laryngological Section of the Royal Society of Medicine by Tilley.[2] In September, 1896, the patient's right vocal cord was removed for epithelioma, the diagnosis being confirmed by histological examination after operation. Since

[1] *Tr. Am. Laryngol. Ass.*, 1909.

[2] *Proc. Roy. Soc. Med.*, Laryng. Sect., 1910, iii, 33.

then the patient enjoyed good health, until November, 1909, when obstructive dyspnœa necessitated tracheotomy, and the patient died a few hours later, aged 78. A well-developed epitheliomatous ulcer occupied the greater part of the left vocal cord.

I have myself recently shown an aged patient to the Bristol Medico-Chirurgical Society in whom a malignant growth of the larynx had existed for two and a half years. The patient was too old to be submitted to operation, as there was considerable glandular involvement when he first came under observation, but he still remains free from pain, and his general health has not been greatly impaired.

Obviously it does not follow that a patient who has malignant disease of the larynx must rapidly succumb unless radical extirpation is performed, and it is well to bear this in mind when the question of submitting to very extensive or dangerous operations have to be considered.

**Treatment of laryngeal tuberculosis by the galvano-cautery.**—Success in this method of treatment of laryngeal tuberculosis has been recorded by many laryngologists. Siebenmann analyses the results in sixty-six phthisical patients treated from 1903 to 1908 in his clinic with laryngeal lesions of varying severity. In the spring of 1909 36 per cent. of these cases were dead, 26 per cent. failed to report, and 38 per cent. reported themselves. Of the latter fourteen cases showed cure, while eleven showed recurrence in the larynx. Siebenmann's technique appears much more severe than that of many operators. A hypodermic injection of morphia is given three-quarters of an hour before operation, and the larynx is sprayed several times with 10 per cent. cocaine-adrenalin solution. The affected areas are then burned widely and deeply through the submucous tissue down to the cartilage, and an attempt is made to complete the operation in one sitting. Ice compresses are applied externally, and the patient put to bed. Œdema of the larynx was common after the operation. In two cases tracheotomy was required, and in one intubation. One patient died of heart disease after the operation, and in another case perichondritis arose leading to fixation of both cords.

We may contrast with the German method the milder, but apparently equally successful method usually adopted in this country. For instance, St. Clair Thomson[2] recently showed two patients: (1) tuberculosis of both vocal processes, cicatrised

[1] *Trans. of Society of German Laryngologists; J. Laryngol.*, 1909, xxiv, 696.

[2] *Proc. Roy. Soc. Med.*, Laryng. Sect., 1909, iii, 6; *J. Laryngol.*, 1909, xxiv, 667.

with seven applications of galvano-cautery between October, 1908, and May, 1909; (2) extensive tuberculosis of the epiglottis, left ary-epiglottic fold, and left inter-arytenoid space. Seven cauterisations were made between February and September, 1909. Improvement was marked from the first, and in November, 1909, when shown, the larynx was soundly healed, although nearly all the epiglottis had been destroyed. Tilley reports a successful case, and regards infiltration cases as the most suitable for this treatment. Dundas Grant also reports several successful cases, and states that the instant relief from pain is the most striking feature.

I have employed galvano-cauterisation in several cases with good results, the most suitable, apparently, being those which show infiltration and tumefaction, especially when there is superficial ulceration. When there is no ulceration, and the tumefaction is localised, I think better results may be obtained by submucous injections; and one of the most striking features of such injections, like that of the galvano-cauterisation, is the remarkable relief to pain. Probably the good effect following galvano-cauterisation is chiefly due to the non-infective inflammatory reaction set up, for it is difficult to suppose that the actual burning due to galvano-cautery punctures can successfully destroy the tubercle bacilli in the whole mass of infiltration.

These methods should be combined with sanatorium treatment and vocal silence, for everything that can be done to assist in the cure must be adopted.

**Röntgen Ray treatment of laryngeal disease.**—Brünings [1] has shown that the rays may be applied in four different ways to the larynx: (1) by internal tubes, the anti-cathode being in the same position as the laryngeal mirror; (2) by endoscopic tubes used through a bronchoscope; (3) by radiation through the skin; (4) by radiation after operation. Only the percutaneous method is generally available, and does not present the difficulties and drawbacks of the other methods, which are still in the investigation stage.

For the percutaneous application it is best to apply the rays from several points consecutively, so as to avoid injury to the skin as far as possible. Hard rays are advisable as absorption is slower, and glass discs should be used as filters to keep back all soft superficial rays. Brünings recommends sittings lasting twenty to thirty minutes, so as to obtain the normal dose of 5 H. when using a focal distance of 20 cm., and tubes of 7 to 8 (Benoist) degrees of hardness.

For the treatment of laryngeal lupus the application of

[1] *J. Laryngol.*, 1909, xxiv, 695.

Röntgen Rays in this manner is worthy of fuller trial, first in order to avoid permanent injury to the vocal cords by operative methods, and to clear up foci of disease which are inaccessible to curettment or galvano-cauterisation.

P. Watson Williams.

---

### Reviews of Books.

